# Climate change and pregnancy complications: From hormones to the immune response

**DOI:** 10.3389/fendo.2023.1149284

**Published:** 2023-04-05

**Authors:** Dennis Yüzen, Isabel Graf, Anke Diemert, Petra Clara Arck

**Affiliations:** ^1^ Laboratory for Experimental Feto-Maternal Medicine, Department of Obstetrics and Prenatal Medicine, University Medical Center of Hamburg-Eppendorf, Hamburg, Germany; ^2^ Institute of Immunology, University Medical Center of Hamburg-Eppendorf, Hamburg, Germany

**Keywords:** climate change, pregnancy, hormones, endocrine system, heat stress, immune system, preterm birth, fetal development

## Abstract

Pregnant women are highly vulnerable to adverse environments. Accumulating evidence highlights that increasing temperatures associated with the ongoing climate change pose a threat to successful reproduction. Heat stress caused by an increased ambient temperature can result in adverse pregnancy outcomes, *e.g.*, preterm birth, stillbirth and low fetal weight. The pathomechanisms through which heat stress interferes with pregnancy maintenance still remain vague, but emerging evidence underscores that the endocrine system is severely affected. It is well known that the endocrine system pivotally contributes to the physiological progression of pregnancy. We review – sometimes speculate - how heat stress can offset hormonal dysregulations and subsequently derail other systems which interact with hormones, such as the immune response. This may account for the heat-stress related threat to successful pregnancy progression, fetal development and long-term children’s health.

## Introduction

**Figure 1:**
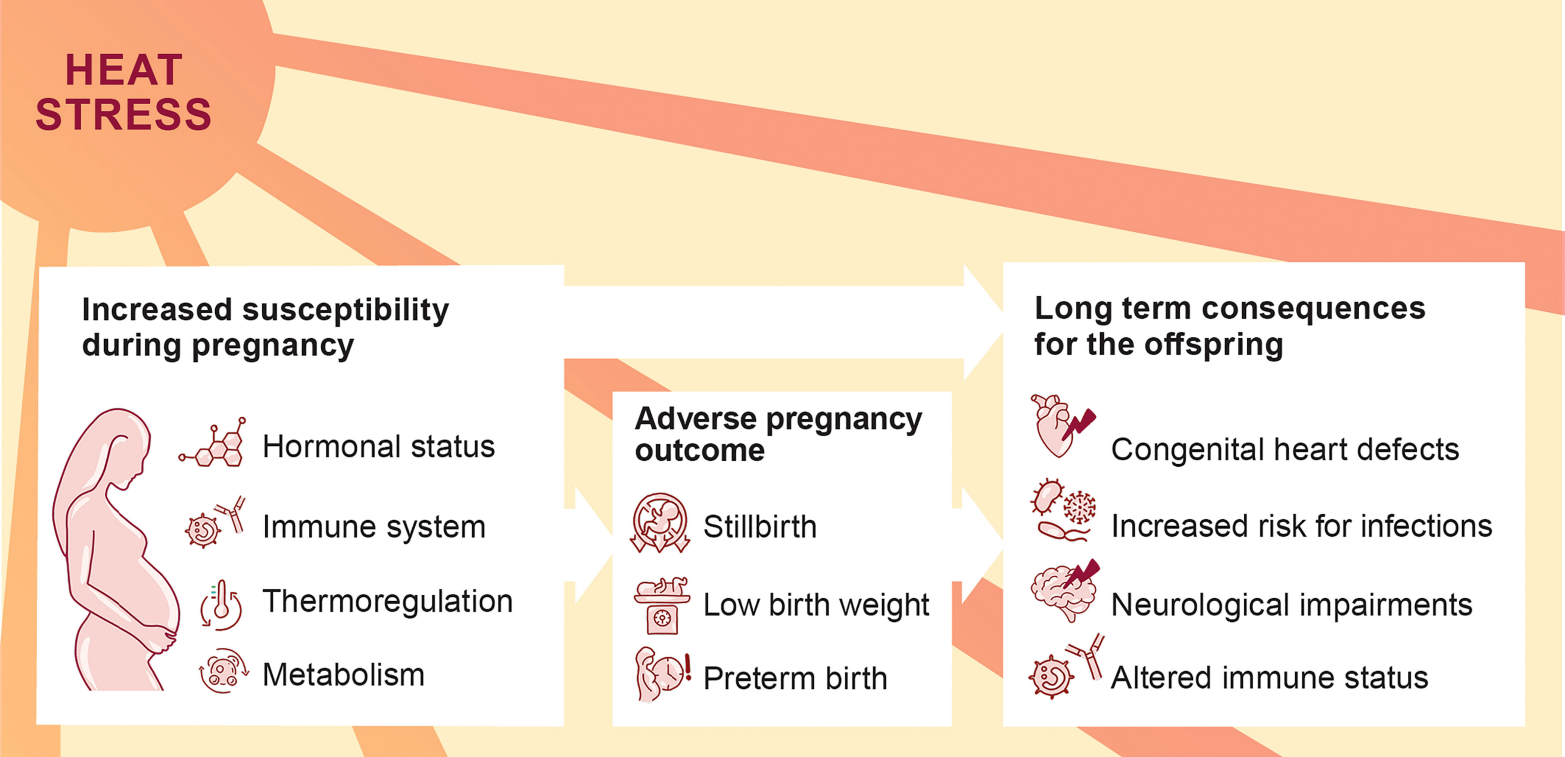
Increased susceptibility of pregnant women to heat stress with possible consequences for the progression of pregnancy and offspring’s health.

Climate change manifests in various environmental threats, including rising temperatures and heat waves. These threats have been associated with severe health consequences, and heat-related illness, *e.g.*, heat cramps, collapse or stroke (1). Pregnant women are highly vulnerable to environmental challenges (2). This vulnerability is attributable to the physiological changes of the maternal cardiovascular and respiratory system, as well as the adaptations of the endocrine and the immune system (**Figure 1**). An increasing number of epidemiological studies provide evidence that environmental heat stress triggers adverse pregnancy outcomes (3, 4). Additionally, heat stress may also interfere with the pre-conceptional phase, resulting in menstrual cycle aberrations and diminished fertility rates (5, 6). Heat stress poses a comprehensive threat to reproductive health with a wide range of personal, societal and socioeconomic consequences.

Successful mammalian reproductive outcome depends on a delicate balance of molecular and cellular markers. A key player is the endocrine system, which not only adapts to the demands of pregnancy by maintaining uterine quiescence, but also adjusts to predictive environmental challenges, e.g., circadian rhythm, as well as unpredicted environmental challenges such as heat stress (7, 8). Dependent on the type or intensity of the challenge, the endocrine system responds with aberrant hormonal levels, which may result in severe consequences for female health (9). The endocrine response is tightly linked to the immune response during pregnancy, through which the immunological tolerance towards the fetus is maintained (10). Therefore, environmental stressors pose a significant risk to disturb the immunological balance essential for feto-maternal tolerance *via* hormone-mediated pathways.

Heat can be defined as temperatures above the thermoneutral zone. The thermoneutral zone allows healthy adults to maintain a physiological body temperature *via* a constant metabolic rate (11). Thus, temperatures above a specific threshold are known to induce heat-related stress responses (12). However, this definitions falls short to take the individual perception of heat - based on the geographical region, acclimatization and personal discomfort - into account. This limitation is also reflected by the heterogeneity of heat stress definitions, exposure windows and exposure duration used in preclinically studies conducted in mice or livestock as summarized for this review in **Table 1**.

Table 1Heat definitions used in the referenced studies considering preclinical models.

**Table:** 

Non-pregnant							
Temp.	RH (%)	Species	Exposure intensity	Exposure duration	Exposure timepoint (gd)	Heat stress definition by author	Ref.
35°C	–	Swine	12 hrs	10 days		–	(13)
38°C	45-60	Rat	360 min/day	98 days		–	(14)
45°C	30	Rat	22-26 min until core temperature = 40°C	Single exposure		Moderate	(15)
			42-48 min until core temperature = 42°C			Severe	
38°C	55	Rat	120 min/day	90 days		Long-term	(16)
Direct solar radiation	–	Cow	420 min/day	~ 21 days		Chronic	(17)
38, 40 and 42°C	50	Mouse	120 min/day	9 days		–	(18)
43°C (abdomen and scrotum)	–	Mouse	15 min	Single exposure		Acute	(19)
32°C	60	Rat	480 min	7 days		Acute	(20)
38°C	–	Rat	60 min	Single exposure		Acute	(21)
38°C	–	Rat	20 and 60 min	Single exposure		–	(22)
42°C	50	Rat	30, 60 or 120 min	Single exposure		Acute	(23)
39°C or 41°C	–	Rat	30 min	Single exposure		–	(24)
42°C	–	Rat	80 min	Single exposure		–	(25)
39°C	50	Mouse	90 min	7, 21 or 42 days		Chronic	(26)
43°C			30 min	Single exposure		Acute	
42°C	50	Mouse	180 min	7, 14, 21 or 28 days		Chronic	(27)
THI>73	–	Cow	All-day	21 days		Acute	(28)

**Table:** 

Pregnant							
Temp.	RH (%)	Species	Exposure intensity	Exposure duration	Exposure timepoint (gd)	Heat stress definition to by author	Ref.
41.2°C	55	Mouse	60 min	Single exposure	14	–	(29)
40-48°C	–	Mouse	60 min	7 days	0.5-5.5, 6.5-14.5, 14.5-17.5	–	(30)
43°C (whole body except head)	–	Rat	15 min	Single exposure	9.5	–	(31)
37-39°C	–	Sheep	All-day	30 days	110	Chronic	(32)
37.5°C (Black globe temp.)	–	Cow	All-day	80 days	160-190	–	(33)
THI>80	–	Cow	All-day	7 or 14 days	103.9	Mild chronic	(34)
40.5°C	–	Mouse	120 min	7 days	1	–	(35)
35°C	–	Mouse	All-day	6 days	12.5	–	(36)

Temp.,Temperature; RH, Relative Humidity; THI, Temperature-Humidity-Index; Ref., Reference; gd, gestational day.

**Figure 2:**
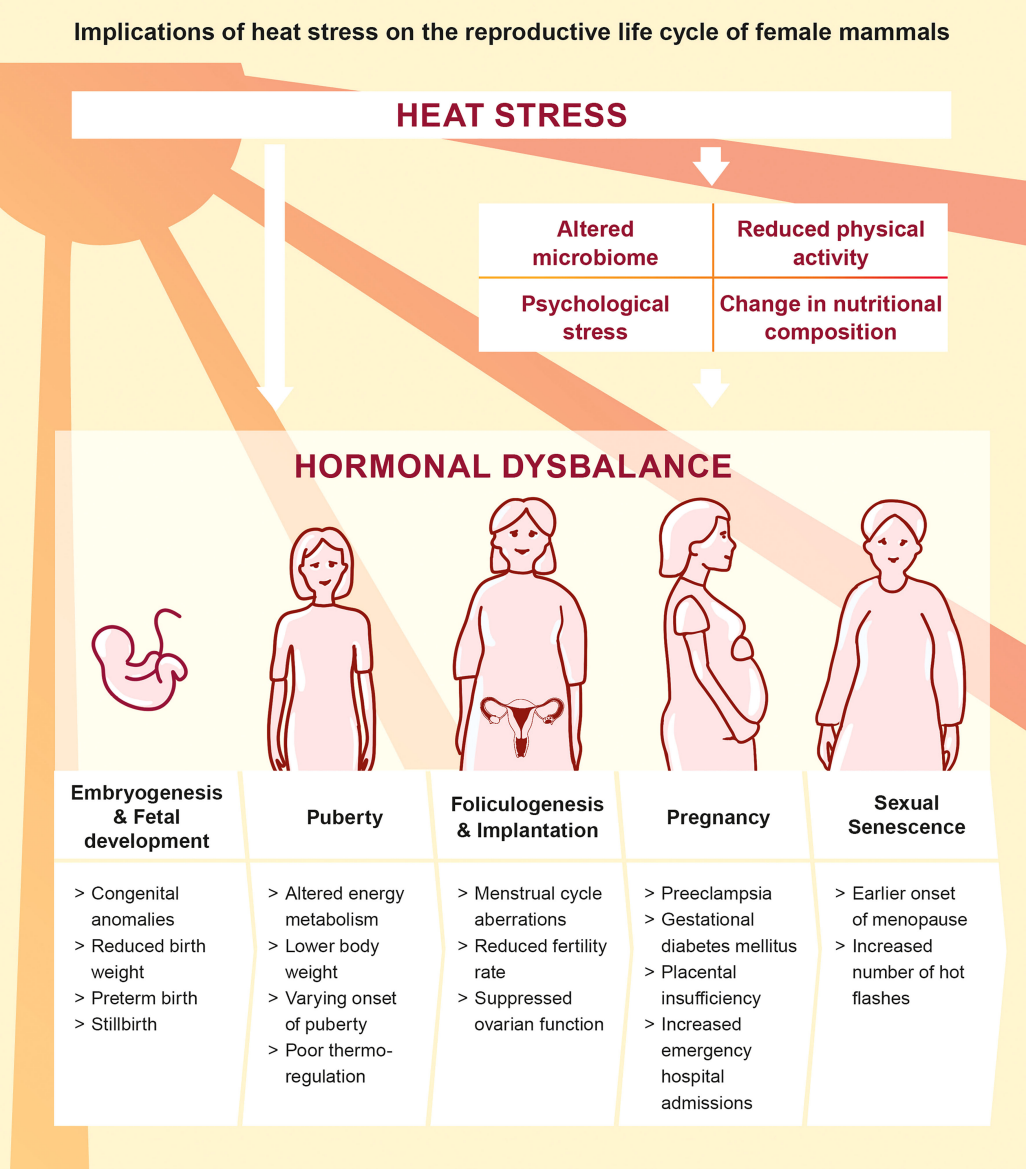
Direct and indirect implications of heat stress on the reproductive life cycle of female mammals (5, 26, 27, 37–45).

The ongoing climate change and the societal responsibility to guarantee maternal health emphasizes the need for in-depth research in order to understand the pathophysiology of heat in the context of female reproduction and hormonal dysbalance throughout women’s life (**Figure 2**). We here summarize recent findings addressing the impact of heat stress on the endocrine and immune system during the preconceptual phase and pregnancy in a number of mammalian species and refine the conceptual understanding on the endogenous stress response to heat stress.

## Heat stress: A threat to reproductive success

Infertility, which is defined as the failure to successfully conceive after more than 12 months of unprotected sexual intercourse, increasingly affects millions of couples worldwide (46). Despite its multifactorial origin, there are several studies indicating that environmental heat can indeed negatively impact fertility in mammals. A study analyzing the effects of high temperatures in the United States between 1931 and 2010 found a decrease in birth rates of 0.4% nine months after exposure to one additional heat day with a mean temperature above 26.7°C compared to an additional day with relatively colder temperatures between 15.6°C and 21.1°C (5). Likewise, a multi-site study revealed that a 1°C increase of the maximum temperature decreases the total fertility rate by 1.3 as shown for Italy, whereas fertility rates of individuals in countries with moderate climate (15-20°C) are unaffected (47). Although heat stress affects both sexes, reduction of reproductive success is partially explained by the observation that heat exposure alters porcine endometrial tissue during the pre-implantation period resulting in diminished fertility rates in females which might be caused by hormonal dysregulation (13). Work done in cattle or rats showed similar results by indicating an association of high ambient temperatures and alterations of the estrous cycle (14, 48). Thus, it is tempting to speculate that the observed reduction in fertility rates upon heat-exposure in women is also caused by alterations of the menstrual cycle. A study investigating the effect of seasonal changes on menstrual cycle length using smartphone application data of 310.000 women failed to support this notion (6). However, this lack in comparability may be explained by the fact that high ambient temperatures and seasonal changes are two different entities.

In addition to the effects of heat stress on women during preconception, exposure to heat has also been associated with the risk for adverse pregnancy outcomes (37). Beside stillbirth, these include preterm birth, low birth weight and congenital cardiac defects, which pose serious threats to the child and can cause long-lasting health impairments (49, 50). Among the possible pregnancy adversities, the risk for preterm birth has likely been best investigated and the majority of studies identified a risk perpetuation caused by heat, as highlighted by a current summary on the topic (51). A meta-analysis of six independent studies reported 1.16-fold higher odds of preterm birth after exposure to extreme heat, along with complications such as lower birthweight, congenital anomalies and stillbirth (3).

## Understanding the pathogenesis of heat stress-induced pregnancy complications: From animal models to humans

Despite the fact that heat-associated pregnancy complications are increasingly in the focus of research endeavors, insights into the underlying pathogenesis are still sparse. This can be partly explained by a general neglect to include pregnant women in research studies. In contrast, the effect of heat stress on livestock has been extensively studied due to its economic importance. Also, cattle are generally kept in controlled environments, which facilitates investigating the effect of environmental stressors. Among mammals, certain milestones of reproduction are conserved, which allows to translate findings on the impact of heat stress in cattle to humans to a certain degree (52). Both species cycle continuously while not pregnant, are monovular **-** ovulation of one oocyte per cycle **-**, have a gestational period of 9 months and have ovaries with a similar size and morphology (53). Additionally, reproductive techniques such as artificial insemination, synchronization protocols and superovulation techniques, to name a few, are widely applied in cattle (54).

In addition to livestock, rodent models can also provide insights on the impact of heat stress on reproductive success, since rodents can be kept in controlled microenvironments. Due to the availability and possibility to generate genetically modified animals, rodents also serve as a powerful source of information to understand the pathogenesis of diseases (55, 56). In the context of pregnancy, mouse models have a high translational value due to a similar placental expression of paternal antigens as well as a comparable immune response (57).

However, large ruminant and small mono-gastric animals deviate with regard to their thermoregulation, which limits the translational value of some of the insights in livestock and rodents. Rodents regulate their body temperature by adapting food intake, ruminants rely on regulation of evaporative heat loss (58, 59). Additionally, the ratio of body surface area and body mass can also affect heat dissipation and thermal regulation (59).

## Effect of heat stress on the hypothalamus-pituitary axis

In humans, heat stress is well known to induce acute neurological deficits and cognitive impairments (60). This may be partly attributed to alterations of the hypothalamic tissue. In rats exposed to severe heat challenges, hypothalamic neurons showed morphological aberrations and an increased frequency of pyknosis (15). Additionally, a moderate to severe heat challenge in rats caused higher levels of oxidative stress. These neuroinflammatory processes were accompanied by an increase in systemic corticotropin-releasing hormone (CRH) and adrenocorticotropin hormone (ACTH) levels, which could be an indicator for increased stress levels and subsequently altered hypothalamic-pituitary hormone secretion. Further, neuroinflammation might also contribute to an impaired temperature regulation and a decrease in heat tolerance (29).

Similar morphological impairments are observed in pregnant mice. Here, acute heat stress led to significantly elevated neuronal damage in the hypothalamus (29). Further signs of hypothalamic cell damage such as pyknosis, cell body shrinkage and apoptotic changes were detected. Similar to the study performed in rats, the authors of the present study also measured increased ACTH levels in heat-challenged pregnant mice. Additionally, morphological alterations were accompanied by significantly higher levels of oxidative stress and increased hypothalamic levels of the pro-inflammatory cytokines TNF-α and IL-1β. These observations not only provide strong evidence for the severe neuronal inflammation upon heat exposure, but also indicates that pregnancy perpetuates these effects. It is known, that the onset of pregnancy alters plasticity and neurophysiological activity of the brain and particularly the hypothalamus (61). These physiological changes might leave the hypothalamic regions more susceptible to heat stress, resulting in further impairment of the adjacent signaling axis. However, increased neuroinflammation during pregnancy is not limited to heat stress, since it was also observed in response to other stressors, such as sound stress or exposure to bacterial antigens (62). In mice, sound stress and bacterial antigens enhanced the permeability of mucosal membranes and subsequent infiltration of bacteria, which led to disseminated inflammation (62). Therefore, the reported consequences of heat stress might be as well attributed to a general stress response during pregnancy.

**Figure 3:**
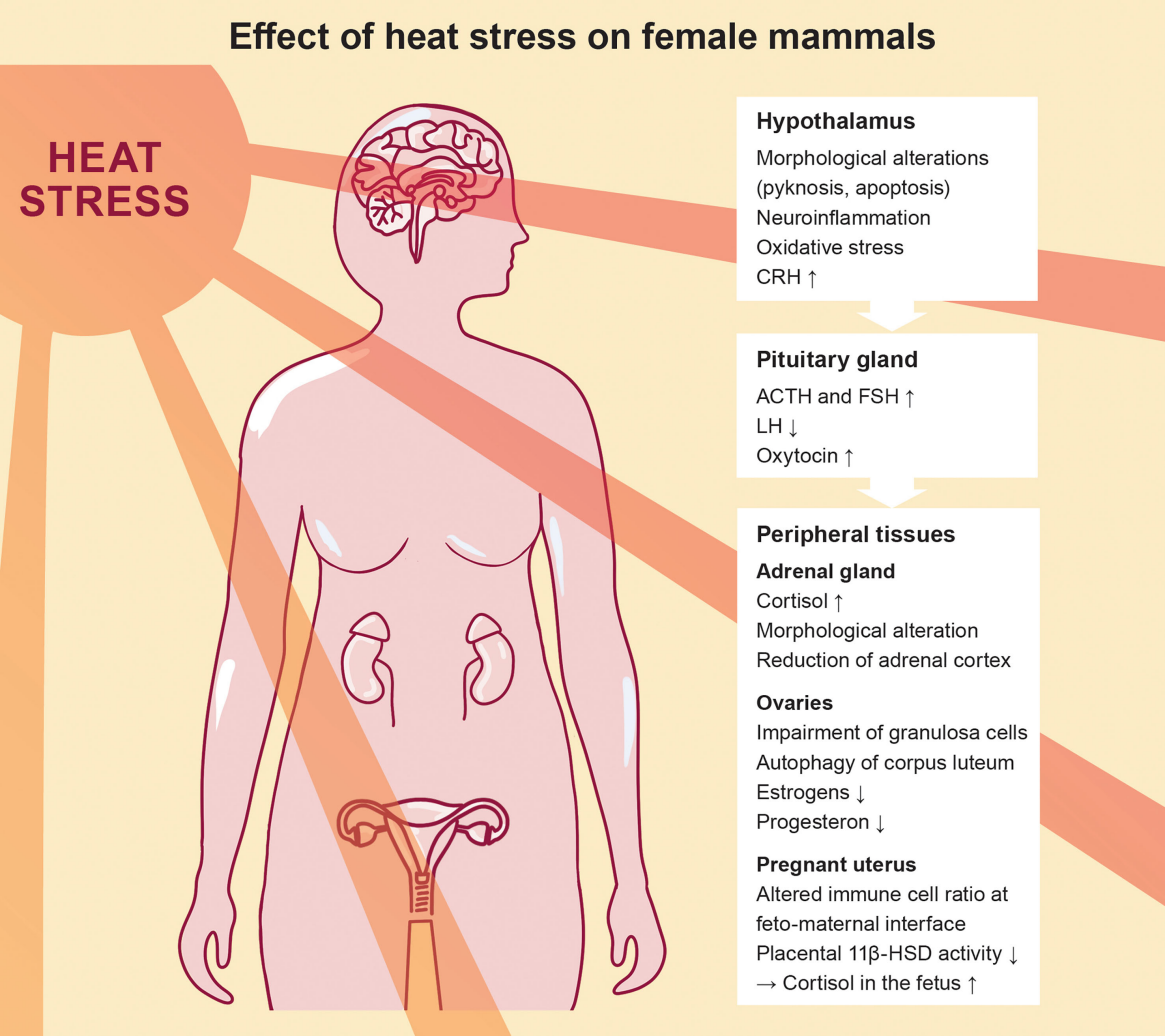
Effect of heat stress on the female mammalian endocrine system, highlighting the progression from the hypothalamus-pituitary axis to the peripheral tissues.

An impairment of the hypothalamic function in response to heat challenge can subsequently affect the secretion of pituitary hormones. In fact, a study performed in rats focusing on gonadotropins identified an increase in follicle-stimulating hormone (FSH) and a decrease in luteinizing hormone (LH) levels in response to heat challenge, whereas gonadotropin-releasing hormone (GnRH) concentrations were unaffected (16). Elevated FSH level upon heat stress have also been observed in dairy cows (17), whereas findings in prepubertal female mice are more conflicting (18). Interestingly, heat stress also caused neuroendocrine perturbations in male mice, mirrored by elevated levels of FSH and reduced levels of inhibin, along with altered reproductive function (19). Taken together, these observations underscore that heat stress directly affects the hypothalamic-pituitary axis, which is summarized in **Figure 3**.

## Peripheral tissues: Adrenal glands, uterus and placenta under heat stress

Physiologically, cortisol or respectively corticosterone levels depend on the secretion by the pituitary and adrenal gland. Both, the pituitary and the adrenal gland are affected by heat stress as indicated by increased organ weights in rats (20). Contradicting results are reported in another study, postulating a decrease in adrenal gland mass and volume attributable to a reduction of the adrenal cortex. More precisely, the alterations can be traced back to a smaller cell size in the zona fasciculata, which is the second of three layers of the adrenal cortex and the main location of cortisol production (21). Both studies differ greatly in the heat exposure protocol used in the experiments. Whereas the first study exposed rats to eight hours of heat stress (32°C) for seven consecutive days, the second study applied 60 minutes of heat stress (38°C) before sacrifice of animals. This opposed experimental design regarding acute or chronic heat exposure combined with the differences in temperatures (**Table 1**) limit comparability of studies and might explain the conflictive findings since moderate and severe heat stress have differential effects on thermoregulation (15).

Based on a comparative study investigating the activation of the HPA axis in rats by various stressors such as fasting, crowding and extreme ambient temperatures, exposure to heat resulted in the highest plasma corticosterone level – the cortisol analog in rodents (22). Both, short periods as well as prolonged exposure to heat led to an increase in blood ACTH and subsequently corticosterone levels (20, 23). Interestingly, evaluation of tissue corticosterone levels provided evidence for differences between peripheral organs after heat exposure, in which the lung-tissue has been proposed as the most accurate indicator for acute heat stress (23). Controversially, a study investigating the effects of heat stress in pre-pubertal rats reported lower corticosterone levels after heat exposure (24), which contradicts the finding that a pre-pubertal state is accompanied by a higher HPA axis reactivity (63, 64). However, the study in pre-pubertal rats assessed corticosterone levels two days after acute exposure to heat, which might already reflect a subsidence of the acute stress response.

Heat-triggered cortisol alterations have not only immediate effects on the maternal health, but might also be associated with longer lasting implications for the offspring. During pregnancy the placenta physiologically produces high amounts of CRH. *Via* a positive feedback on the maternal HPA axis, this results in a 40 fold higher CRH production from first trimester to term and a 3-5 fold rise in cortisol over gestation (65). Due to its properties as a steroid hormone, cortisol possesses the ability to cross the feto-maternal barrier. Thus, maternal cortisol levels affect the developing fetus. To protect the fetus from these high levels of maternal cortisol, the placenta functions as an important metabolizer (66). Placental metabolism of cortisol is mainly regulated by the enzyme 11-beta-hydroxysteroid dehydrogenase 2 (11ß-HSD2), which converts cortisol to a biologically inactive form (67). During heat stress in pregnant mice, expression of 11ß-HSD2 was reduced, along with an expected increase of cortisol in the fetus (68). This suggests that alterations of placental function – a key endocrine organ during pregnancy – may expose the fetus to cortisol surges upon heat stress. Changes in placental function might be attributed to heat-specific reductions of placental growth or to an altered HPA axis, since variations in 11β-HSD expression have also been reported upon other stress challenges in mice (30, 69). These cortisol surges can cause long-lasting health consequences for the offspring, resulting in growth retardation of the adrenal cortex and a decreased population of somatotropes – growth hormone producing cells – in the adenohypophysis (31) or a larger volume of the right amygdala associated with affective problems in girls (70).

In addition to cortisol, placental trophoblast cells produce human chorionic gonadotropin (hCG) at the beginning of pregnancy. hCG has a luteinizing effect on ovarian cells, which lengthens the lifespan of the corpus luteum and thus leads to sustained production of progesterone. At the moment there are no studies addressing the effect of heat exposure on hCG secretion.

The derogation of successful reproduction caused by heat stress does not only manifest through placental alterations, but might also be linked to heat-triggered effects on the uterus. In fact heat stress has been shown to affect the morphology of the preimplantational endometrium, especially affecting the luminal epithelial cells, and triggering aberrant local cell proliferation as well as the dilation of the uterine glands (16). During pregnancy the maintenance of uterine quiescence could be interrupted by increased FSH concentrations acting on the myometrium potentially favoring preterm births (71). Another hormone highly relevant for the induction of labor is oxytocin. Produced in the hypothalamus, oxytocin mediates, beside its anti-inflammatory and anti-apoptotic functions, uterine contraction during birth. Pretreatment of rats with oxytocin before the onset of heat stress protected them from heat stroke related symptoms such as lung edema and increased over-all well-being (25). In line with these protective capacities of oxytocin before exposure to heat, oxytocin concentrations are increased during heat stress (32). This suggests an acute protection mechanism, mediated by its effects on vascular resistance, cardiac output or the overall induction of a hypotensive response (72).

## Heat stress modulates sex hormone levels

Several studies reported some form of dysfunction in the female gonads upon heat exposure. Irregular phenotypes of granulosa cells accompanied by detachment of oocytes from the granular cell layer could be found in acute and chronic heat stressed mice (26, 27). Consistent with the morphological changes of the granulosa cells, a decrease in estradiol concentrations upon chronic heat stress has been observed (16, 27, 73). This might be explained by decreased expression patterns of the enzyme aromatase in response to heat stress, which is the key hormone of the estrogen synthesis (27). Despite granulosa cell damage, gonadotropin receptors on the cell surface show diminished FSH receptor expression upon acute heat stress in rats, along with increased FSH receptor expression in chronic heat stressed rats (16, 74). However, the exact pathways by which heat stress influences morphological integrity and receptor expression of granulosa cells are unknown. Further, it needs to be investigated why granulosa cells seem to be more susceptible to heat-related damages compared to other cell types. The relevance of heat as a stressor was demonstrated by a study comparing estradiol levels after heat or psychological stress. Although exposure to both stressors lowered estradiol levels, this effect was more pronounced after heat stress. Interestingly, combination of both resulted in even lower levels of estradiol indicating cumulative effects on gonadal tissue damage (14).

Additionally, considering the fact that regulation of the hypothalamic-pituitary-gonadal (HPG) axis functions *via* negative and positive feedback mechanisms, low hormone concentrations due to impaired production by the gonadal tissues have been proposed to lead to a decreased feedback response in the hypothalamus and the pituitary gland and stimulate the secretion of gonadotropins (75).

Although estrogens are pivotal during pregnancy, few studies to date focused on the dynamics of estrogen levels throughout mammalian pregnancy in response to heat stress. One study dating back to 1982 failed to detect any heat-related alterations of free estrogens in chronic heat stress-exposed pregnant cows (33). However, the authors describe a decrease of estrone-sulfate upon heat stress, which indicates placental insufficiency. Since the placenta is the major source of estrogens during human pregnancy, future studies evaluating estrogen levels should therefore also consider placental functionality. Also, changes in estradiol levels might be particularly relevant in the context of thermoregulation, since estradiol promotes vasodilatation and thus facilitates heat dissipation lowering the body temperature (76, 77). Thus, a comprehensive study addressing possible changes of estrogens in combination with placental assessment might provide novel insights on the consequences of heat stress.

Besides its pivotal role in establishing fertility and pregnancy maintenance, progesterone is also a thermal influencer. In contrast to estrogen, progesterone promotes heat conservation and higher body temperature (76). An increase in progesterone, as evident during pregnancy, might thus explain the high susceptibility to heat stress due to an inability to dissipate heat, subsequently favoring pathological hyperthermic conditions. Although, the exact contribution of progesterone to thermoregulation is not fully understood, studies in rodents provide evidence that chronic heat stress resulted in reduced concentrations of progesterone (14). This observation was also confirmed in heat-stressed cattle (34). However, this effect was no longer evident in rats exposed to heat if challenged for a prolonged period of time (16). Reduction in progesterone levels might be linked to a decreased synthesis of pregnolone, the precursor of progesterone, which was diminished in bovine granulosa cells due to a decreased transcription of steroidogenic genes (78). However, to date there are no data available on how heat exposure alters the expression of steroidogenic genes in murine or even human cell culture experiments.

Comparable to the findings in non-pregnant mice, heat stress exposure of early pregnant mice led to a significant decrease in progesterone concentrations (35). Concurrently to these hormonal changes, increased markers of autophagy were detected in the steroid producing cells of the corpus luteum. Autophagy is relevant for regulating the function of the corpus luteum and might be relevant for luteal regression. Since progesterone is produced by the corpus luteum at the beginning of pregnancy, before the placenta takes over, regression by autophagy could contribute to decreased progesterone concentrations. This hypothesis is supported by observations in chronic heat stress-exposed cattle three weeks before gestation, where lower progesterone levels, reduced conception rates and lower transferable embryo were described (28).

Another explanation relates to the common progenitor of progesterone and cortisol: cholesterol-derived pregnenolone. Increased demand of cortisol in response to heat stress could lead to a competition of both syntheses and reduce progesterone availability (66).

## Heat stress and the immune system during pregnancy

During the course of pregnancy, the maternal immune system undergoes gradual adaptations, which are essential to guarantee maternal tolerance towards the genetically foreign fetus. Dysregulation of these immunological adaptations is associated with pregnancy complications, such as recurrent spontaneous abortions, preterm birth or intrauterine growth restriction (79). Hormones significantly orchestrate the maternal immune adaptations during pregnancy (57). Thus, heat stress-induced alterations of the endocrine response can also affect the immune response in pregnant individuals. Progesterone primarily dampens the production of pro-inflammatory cytokines by local immune cells and induces more tolerogenic phenotypes in dendritic cells, which then enables them to promote regulatory T cells, essential for pregnancy maintenance (80, 81). In addition, progesterone also promotes immune tolerance by inducing immunomodulatory molecules at the feto-maternal interface (82). As discussed above, heat exposure seems to reduce progesterone concentrations. A decrease in progesterone might subsequently lead to a shift towards an inflammatory immune response at the beginning of pregnancy, with decreased tolerance towards the fetus, which then favors fetal loss (62). Towards the end of human pregnancy, progesterone contributes to maintain an anti-inflammatory environment until induction of labor. Thus, heat stress associated progesterone reduction might trigger inflammation processes prematurely and contribute to the pathogenesis of heat-induced preterm birth.

Glucocorticoids such as cortisol are well known for their anti-inflammatory capacity. Additionally, they are involved in cell recruitment of macrophages to the feto-maternal interface as demonstrated in experiments with glucocorticoid receptor knock-out mice (83). Although the exact function of glucocorticoids in the context of the initiation of labor has yet to be elucidated, it is well known that in addition to the increase of glucocorticoid levels during the course of pregnancy a significant surge takes place when approaching labor (84). Thus, increase of glucocorticoid levels – as observed upon heat exposure – might not only contribute to an impaired immune response early during pregnancy, but also towards term. Studies evaluating the immune cell function at the feto-maternal interface upon heat stress triggered hormone alterations are missing and should urgently be conducted starting with preclinical models. Due to the immunological dynamics of pregnancy, different exposure windows should be tested and clearly defined to assure reproducibility.

Studies testing the direct effect of heat stress on immune cells during pregnancy are sparse. One study evaluating placental immune response to heat stress in late gestational mice described an increase in the expression of inflammatory genes (36). Here elevated molecular expression of the macrophage marker CD68 were accompanied by an upregulation of genes associated with the complement system, such as C1qa, C3 and CD55. The authors hypothesize that heat stress led to a recruitment of macrophages to the placenta, which then activate the complement system. Additionally, a decrease of Csf1was observed. Csf1 can induce an anti-inflammatory phenotype in macrophages, suggesting that its decrease in response to heat stress skews macrophage function towards pro-inflammation. Macrophages have pleiotropic functions during pregnancy, therefore a possible change in function in response to heat stress must be confirmed in future studies.

Additionally, the immune system may not only be acutely affected by heat stress, since long-term consequences have also been postulated. Triggered by the acute stress response, heat stress may lead to an acclimatization that enhances cytoprotective anti-oxidative and anti-apoptotic pathways against future stress exposure (85). This mechanism, referred to as heat acclimation-mediated cross-tolerance, is mediated by epigenetic modification of histones, namely phosphorylation of histone H3 and acetylation of histone H4, which promote gene expression of cytoprotective mediators (86, 87). Since these observations were made in male rats, they urgently require further validation in pregnancy models.

## Psychological consequences of heat stress: From sleep deprivation to climate anxiety

In addition to the discussed direct effects of heat exposure on hormone levels or immune homeostasis, heat may also indirectly modulate the endocrine pathways of HPG axis. Tropical nights (night-time minimum temperature equal or higher than 20°C) (88) are associated with sleep deprivation and reduced quality of sleep. Both have been linked to alterations of sex hormone concentrations, which negatively impact reproductive fitness in rats (89, 90). Here, the crosstalk between reproductive hormones and melatonin is of great interest, since sleep deprivation is known to impact the endogenous melatonin secretion. Melatonin modulates sex hormone synthesis especially in the hypothalamus and the pituitary gland (7). For the years 2050 and 2099, the predicted numbers of additional nights of insufficient sleep with 2010 as baseline average are estimated to be 6 respectively 14 per 100 individuals (90). Thus, the relevance of sleep deprived hormonal imbalances in the near future should not be underestimated.

Climate change poses a direct threat to mental health of pregnant women by favoring adverse pregnancy outcomes. Acute environmental events such as hurricanes, droughts or wildfires, and also chronic exposure to increased air pollution and high temperatures leads directly to mental health disorders such as posttraumatic stress syndrome or depression and indirectly by favoring water and food insecurities as well as migration (91). Recently, climate anxiety has been introduced as additional mechanism on how climate change effects mental well-being. Climate anxiety describes distress about the consequences of climate change, the existential threat and uncertainty that climate change poses, which cannot be anticipated neither in time nor place (92). Interestingly, this anxiety is strongly linked to the individual perception of climatic changes and does not necessarily correspond to personal experiences, since it includes individuals, who have not experienced climate-related adverse events. Climate anxiety itself is not pathophysiological, but poses a permanent psychological stressor and bares the potential to become chronic and thus, clinically evident. Especially adults at the beginning of their reproductive years reported to be extremely worried about climate change (93), which even results in a hesitancy to have children. Given the high prevalence of these negative emotions and beliefs concerning the future of families and children, climate anxiety constitutes a significant psychological stress factor that must be urgently evaluated in female and male adults during their reproductive years.

In contrast to high temperatures, which directly affect thermoregulation, the indirect effect of heat in inducing an endogenous stress response – mirrored by heat-induced sleep deprivation and climate anxiety – must be taken into account, since this enhanced stress perception perpetuates the endocrine stress response in pregnant as well as non-pregnant individuals.

## Outlook

**Figure 4:**
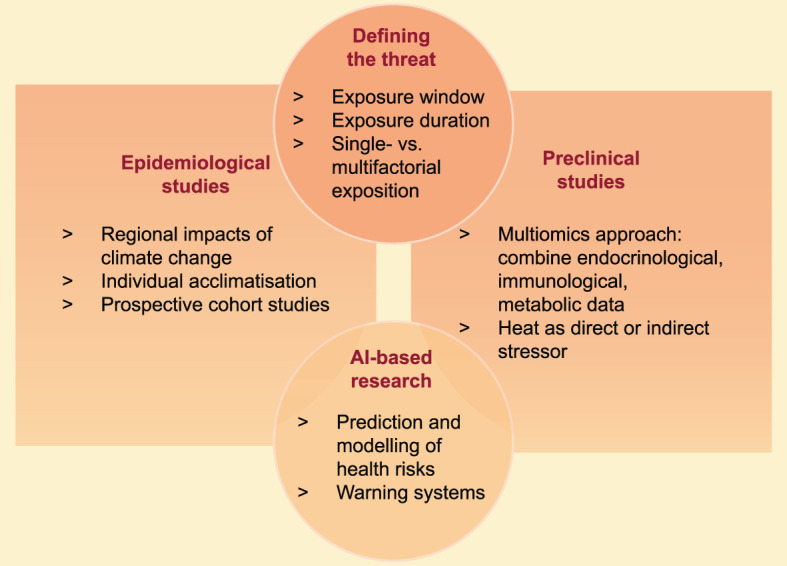
Future directions of heat stress related pregnancy research.

This review highlights the multifaceted consequences of heat stress on the endocrine balance and immune homeostasis, especially in the context of pregnancy. Clearly, many open questions remain before we fully understand how heat stress affects reproductive outcome. E.g., it remains to be tested if heat exposure directly affects the thermoregulation in pregnant women *via* inducing cell stress, hereby inducing endocrine and immunological aberrations. Or whether heat stress simply functions as an external stressor, which activates the endogenous stress response, hereby activating the hormonal stress response cascade. In fact, both pathways can alter fetal and childhood development trajectories and affect the endocrine hemostasis of this next generation. Also, the impact of acute *vs*. chronic heat stress effects remains to be elucidated, and multifactorial risk assessments should be considered in order to control for possible confounder of heat stress, such as ozone and air pollution. The limited number of studies addressing these observations in animal pregnancy models or human pregnancy cohorts leave a blind spot to the vulnerable societal group of pregnant women. Given the rapid progression of climate change and cumulating weather extremes, it is of particular importance to intensify efforts of local research groups and global initiatives. However, to address these knowledge gaps, universal definitions of chronic and acute heat stress need to be established, to allow for comparison between studies (**Figure 4**). Given the increasing number of studies reporting on pregnancy adversities due to environmental heat stress on a global scale, these observations demand an interdisciplinary expertise from clinicians, lab scientists, statisticans and epidemiologists.

## Author contributions

All authors contributed to the article and approved the submitted version.
